# Editorial: Cytoprotective role of mitochondria in reproduction

**DOI:** 10.3389/fendo.2024.1448126

**Published:** 2024-08-05

**Authors:** G. Jaita, S. P. Kodithuwakku

**Affiliations:** ^1^ Instituto de Investigaciones Biomédicas (INBIOMED), Facultad de Medicina Consejo Nacional de Investigaciones Científicas y Técnicas (CONICET)- Universidad de Buenos Aires, Buenos Aires, Argentina; ^2^ Departamento de Biología Celular e Histología, Facultad de Medicina-Universidad de Buenos Aires Buenos, Buenos Aires, Argentina; ^3^ Institute of Veterinary Medicine and Animal Sciences, Etonian University of Life Sciences, Tartu, Estonia; ^4^ Department of Animal Science, Faculty of Agriculture, University of Peradeniya, Peradeniya, Sri Lanka

**Keywords:** mitochondria, reproduction, infertility, ovarian insufficiency, oocyte, Sertoli cell, embryo development, stress response

Mitochondria, often hailed as the powerhouse of the cell, have transcended their traditional role of energy suppliers to emerge as pivotal players in reproductive biology. This editorial delves into the cytoprotective mechanisms of mitochondria that safeguard reproductive processes.

Mitochondria are integral to energy production, but their role in reproduction is multifaceted. They regulate germ cell fate and ensure the lineage’s continuity, acting as gatekeepers of cellular integrity plus recent findings indicate a role in vertical genetic material transfer from mother to embryo (1). In the reproductive milieu, mitochondria are the unsung heroes, shielding cells from oxidative stress and other insults. Their ability to modulate the cellular environment ensures the preservation of reproductive tissues against age-related degeneration. The discovery of Mitochondria-Derived Peptides (MDPs) like Humanin has opened new avenues in reproductive cytoprotection (2). These peptides are potent antioxidants, offering a new layer of defense against cellular stressors. Mitochondrial dysfunction can be a harbinger of compromised fertility. It was reported that the mitochondrial DNA copy number in oocytes, cumulus granulosa cells, and peripheral blood could be biomarkers of reproductive potential, reflecting the intricate link between mitochondrial health and fertility (3–5). Targeting mitochondrial pathways could offer promising therapeutic strategies for reproductive diseases. The modulation of these pathways could revolutionize treatments for conditions like ovarian aging and infertility.

This Research Topic included studies covering different areas where mitochondrial functionality plays a predominant role in reproduction. Mitochondrial function’s impact is crucial in physiological and pathophysiological ovarian conditions. Primary ovarian insufficiency (POI) represents a prevalent condition within the dominion of gynecology, characterized by profound consequences that encompass a diminished probability of pregnancy and the manifestation of symptoms associated with low estrogen levels. Traditional Chinese Medicine (TCM) has been acknowledged as a potent intervention for addressing POI within the scope of outdated therapeutic approaches. Nevertheless, the intricacies underlying the efficacy of TCM in this context remain enigmatic. Exploring this research domain, Miao et al. have shown findings that suggest a notable improvement in ovarian functionality in mice afflicted with POI. In the context of POI, the inhibition of PINK1-Parkin mitophagy and NLRP3 inflammasome activation by TCM could potentially prevent excessive inflammation and mitochondrial dysfunction, which are thought to be contributing factors to the pathology of POI. By modulating these pathways, TCM may enhance ovarian function and ameliorate the symptoms associated with POI. However, further research is needed to fully elucidate these mechanisms and their implications for the treatment of POI.

On the other hand, in reproductive health, obesity has emerged as a significant concern due to its detrimental effects on fertility, accompanied by a spectrum of metabolic complications that have garnered widespread attention. The convergence of metabolomics and lipidomics stands at the forefront of innovation, heralding a new era in the evolution of precision medicine. The study by Xu et al. presented a compelling narrative on the regulatory role of palmitic acid, a saturated fatty acid, in the metabolic processes of Sertoli Cells, which are pivotal for male fertility. By combining the analytical power of metabolomics and lipidomics, the researchers wanted to illuminate the potential pathways through which palmitic acid exerts its effects. Their findings indicate that palmitic acid causes a decline in mitochondrial efficacy, impeding the cells’ ability to perform fatty acid β-oxidation—a critical metabolic route for energy production. This metabolic disruption culminates in the initiation of apoptosis, the programmed cell death, which carries a danger to the viability and correct functioning of Sertoli Cells. The implications of this study are profound, offering novel insights into the molecular dynamics of lipotoxicity. It underscores the necessity for a deeper understanding of how dietary components, such as palmitic acid, can influence cellular metabolism and contribute to conditions that affect fertility. Furthermore, the research underscores the potential for targeted interventions that could mitigate the adverse effects of lipotoxicity on Sertoli Cells, thereby enhancing reproductive health outcomes.

Also, the present Research Topic contemplated the study of exosome mitochondrial function in human follicular fluid to understand about embryo development. Follicular fluid is a complex biological fluid that contributes to the micro-environment of oocyte development. Yu et al. evaluated the role of steroid and gonadotropic hormone levels and mitochondrial function in embryo development during *in vitro* fertilization cycles. The levels of follicular fluid steroid and gonadotropic hormones from a single follicle can predetermine subsequent embryo development to some extent. In addition, the authors describe that impaired exosome mitochondrial dysfunction can be a potential factor that causes hormone change during the embryo development.

Within the pregnancy, preeclampsia is one of the leading causes of maternal and fetal morbidity and mortality worldwide. Preeclampsia is linked to mitochondrial dysfunction as a contributing factor in its progression. Another study in this Research Topic by Huang et al. aimed to develop a novel diagnostic model based on mitochondria-related genes (MRGs) for preeclampsia using machine learning and further investigate the association of the MRGs and immune infiltration landscape in preeclampsia. This study has provided a novel diagnostic model and genes for preeclampsia while revealing the association between MRGs and immune infiltration. These findings offer valuable insights for further research and treatment of preeclampsia.

Reproduction is also affected by oncology treatment as chemotherapy which is extensively used to treat cancers and is often associated with ovarian damage and leads to premature ovarian insufficiency and infertility. However, the role of mitochondria during ovarian damage with chemotherapy remains unknown. This Research Topic includes the study from Li et al. which used a mouse model with oocyte-specific deletion of mitochondrial stress response gene Caseinolytic peptidase P (*Clpp*) to investigate mitochondrial homeostasis in oocytes from mice receiving chemotherapeutic drug cyclophosphamide (CTX). The authors indicate that ClpP is required for oocyte competence during maturation and early folliculogenesis, and its deficiency deteriorates cyclophosphamide-induced ovarian damage.

Taken together, all the studies in this Research Topic contribute substantially to the knowledge of the role of mitochondrial function as part of the mechanisms involved in the cytoprotection of reproduction. Thus, paving the path for more in-depth studies for future medical interventions to improve human reproduction specially under current low fertility rates in human.

## Author contributions

GJ: Conceptualization, Visualization, Writing – original draft, Writing – review & editing. SK: Conceptualization, Visualization, Writing – original draft, Writing – review & editing.
